# Risk factors for acute bilirubin encephalopathy on admission to two Myanmar national paediatric hospitals

**DOI:** 10.1186/s40748-015-0024-3

**Published:** 2015-09-15

**Authors:** G. Arnolda, H. M. Nwe, D. Trevisanuto, A. A. Thin, A. A. Thein, T. Defechereux, D. Kumara, L. Moccia

**Affiliations:** Thrive Networks, Oakland, CA USA; School of Public Health & Community Medicine, Faculty of Medicine, University of New South Wales, Wales, NSW Australia; Department of Paediatrics, University of Medicine (1), Yangon, Myanmar; Amici della Neonatologia Trentina, Trento, Italy; Children and Women’s Health Department, Medical School University of Padua, Padua, Italy; Mandalay Children’s Hospital (300), Mandalay, Myanmar; Department of Neonatology, University of Medicine (1), Yangon, Myanmar; Department of Surgery, Liege University Hospital, Liege, Belgium

**Keywords:** Neonatal jaundice, Hyperbilirubinaemia, Acute Bilirubin Encephalopathy, Kernicterus, Chronic Bilirubin Encephalopathy

## Abstract

**Background:**

Jaundice is the commonest neonatal ailment requiring treatment. Untreated, it can lead to acute bilirubin encephalopathy (ABE), chronic bilirubin encephalopathy (CBE) or death. ABE and CBE have been largely eliminated in industrialised countries, but remain a problem of largely undocumented scale in low resource settings.

As part of a quality-improvement intervention in the Neonatal Care Units of two paediatric referral hospitals in Myanmar, hospitals collected de-identified data on each neonate treated on new phototherapy machines over 13–20 months. The information collected included: diagnosis of ABE at hospital presentation; general characteristics such as place of birth, source of referral, and sex; and a selection of suspected causes of jaundice including prematurity, infection, G6PD status, ABO and Rh incompatibility. This information was analysed to identify risk factors for hospital presentation with ABE, using multiple logistic regression.

**Results:**

Data on 251 neonates was recorded over 20 months in Hospital A, and 339 neonates over 13 months in Hospital B; the number of outborn neonates presenting with ABE was 32 (12.7 %) and 72 (21.2 %) respectively. In the merged dataset the final multivariate model identified the following independent risk and protective factors: home birth, OR_adj_ = 2.3 (95 % CI: 1.04-5.4); self-referral, OR_adj_ = 2.6 (95 % CI: 1.2-6.0); prematurity, OR_adj_ = 0.40 (95 % CI: 0.18-0.85); and a significant interaction between hospital and screening status because screening positive for G6PD deficiency was a strong and significant risk factor at Hospital B (OR_adj_ = 5.9; 95 % CI: 3.0-11.6), but not Hospital A (OR_adj_ = 1.1; 95 % CI: 0.5-2.5).

**Conclusion:**

The study identifies home birth, self-referral and G6PD screening status as important risk factors for presentation with ABE; prematurity was protective, but this is interpreted as an artefact of the study design. As operational research, there is likely to be substantial measurement error in the risk factor data, suggesting that the identified risk factor estimates are robust. Additional interventions are required to ensure prompt referral of jaundiced neonates to treatment facilities, with particular focus on home births and communities with high rates of G6PD deficiency.

## Background

High levels of unconjugated bilirubin in the neonate can lead to the development of acute bilirubin encephalopathy (ABE) which first presents as lethargy, hypotonia and poor sucking. If untreated this can proceed to hypertonia manifested as backward arching of the neck and back (retrocollis and opisthotonos), and ultimately to apnoea, coma and death [1]. The long term sequelae of bilirubin toxicity, termed chronic bilirubin encephalopathy (CBE), previously ‘kernicterus’ [2], is characterised by a combination of abnormal motor control, movements and muscle tone, disturbed auditory processing, impairment of upward vertical gaze, and dysplasia of the enamel of deciduous teeth [3].

In high resource settings, the incidence of CBE decreased markedly with the introduction of double volume blood exchange transfusion in the 1940s [4]. The need for exchange transfusion, in turn, has been reduced by the use of phototherapy [5], post-partum administration of anti-D immune globulin G to Rh(D) negative mothers exposed to an Rh(D) positive fetus to prevent maternal Rh (D) alloimunisation that can lead to neonatal haemolytic disease in future pregnancies [6], or immunoglobulin directly to the newborn as treatment for Rh (D) or ABO haemolytic disorders [7].

A variety of estimates of the incidence of CBE in high resource settings suggest that the incidence in the early twenty-first century ranges from 1.0-3.7 per 100,000 live births [8]. The incidence increases with Total Serum Bilirubin [TSB]; of neonates with TSB >428 μmol/L, about 6 % develop CBE, while of neonates with TSB >513 μmol/L, around 14 % develop CBE [8].

In low resource settings, information about the actual incidence of ABE and CBE is sparse. A population estimate is available in a survey of 16,979 people <20 years old in Kolkata, India, which found a prevalence of cerebral palsy [CP] of 283/100,000, with 16.7 % of the CP associated with a history of ‘profound jaundice’ (i.e., 47 cases of jaundice-related CP per 100,000 live births) [9]; this prevalence of jaundice-related CP is an underestimate of the actual incidence of CBE to the extent that there is excess mortality among children and young people with jaundice-related CP. A separate population estimate derived from a four-month study in a single Baghdad hospital, estimated an incidence of ABE of 1,749/100,000 live births, during a period of severe health system disruption in 2007–08 [10].

Despite our lack of detailed understanding of the mechanism for bilirubin neurotoxicity, experience in industrialised countries makes clear that we have sufficient knowledge and technical capacity to virtually eliminate CBE. A systems-based approach has been recommended in high resource settings, incorporating individual risk assessment and/or pre-discharge TSB, lactation support, close-follow up in the community, and prompt and effective intervention when needed [11]. Improving systems of care in low-resource settings, along similar lines, is feasible.

The data presented in this report were collected in the course of a pilot project to improve care in the Neonatal Care Units (NCUs) at two specialist paediatric referral hospitals in Myanmar. As neonatal jaundice was the most common reason for admission to the target NCUs, and to other NCUs not included in the pilot project, we undertook to collect relevant information as part of program implementation, to better understand the clinical spectrum of jaundiced neonates at admission, to guide project enhancement in the target hospitals, and to inform the future direction of jaundice prevention and treatment programs in Myanmar. These data, while primarily collected for the purpose of planning and project evaluation, were retrospectively examined to identify pre-admission risk factors for admission to hospital with ABE.

## Methods

### Setting and context

This study was conducted as part of an intervention at two tertiary paediatric referral hospitals in Yangon and Mandalay, Myanmar, hereafter referred to as Hospitals A and B. We chose these institutions because, in late 2011, they were the two most significant national level tertiary paediatric referral hospitals in Myanmar; neither hospital provided birthing facilities, but both had a NCU for neonatal admissions. The data reported here were initially collected for a separate purpose – to monitor and evaluate the intervention. In the current study, we have used that data opportunistically, to identify risk factors for ABE at hospital admission and to guide the design of research and future interventions to encourage earlier hospital presentation of neonates at risk of ABE.

### Ethical approval

Data were collected in the process of routine care provision. De-identified data were originally collected for operational purposes, but once a decision to publish was made, retrospective ethics clearance was sought from the Ethical Committee on Medical Research involving Human Subjects, Myanmar Department of Health [approval #14/2014].

### Data collection

During the training, both hospitals agreed to collect data on each neonate treated on the LED phototherapy machines donated as part of the intervention, and participated in the specification of the data items for an LED Phototherapy Treatment Register (LPTR). Hospitals were not required to collect information on neonates treated exclusively with conventional fluorescent light phototherapy machines. While the data elements were prospectively defined, we decided to explore risk factors for presentation with ABE retrospectively, so the study is best described as a retrospective cohort.

Individual patient information collected in the LPTR included the following variables: hospital name; date of birth; gender; gestational age; admission weight; place of birth (home/health facility); source of referral (self/health facility); signs of kernicterus, the term routinely used in Myanmar to describe both ABE and CBE, at admission, classified as ABE (Yes/No); previous sibling had received phototherapy (Yes/No); significant bruising (Yes/No); breastfeeding + feeding poorly (Yes/No); other suspected cause of jaundice (sepsis/G6PD/ABO/Rh D/Other/Not stated; multiple selections permitted); date started phototherapy on LED machine; TSB at start of phototherapy; TSB prior to exchange transfusion; and discharge status (discharged [with or without ‘kernicterus’]/removed by family/transferred/died).

TSB readings were performed in the hospital laboratory at Hospital A. At Hospital B, the overwhelming majority of readings were performed in the NCU on a desktop Bilirubinometer with a maximum of 513 μmol/L; some readings were performed in the hospital laboratory, but we did not record which tests were performed in which location.

At both facilities, G6PD screening was performed using the qualitative methaemoglobin reduction test. Although G6PD deficiency is an X-linked genetic disorder, both hospitals screen all neonates admitted for treatment of jaundice, not just males. At the time of the study, families were expected to pay for the test, but hospitals paid if families could not; clinicians are therefore confident that the vast majority of eligible neonates were screened. While this G6PD screening test is adequate in most situations, it is known to produce false-negative results in neonates with low to moderate levels of G6PD deficiency who are haemolysing, so the test must be repeated 2–4 weeks later to determine actual G6PD deficiency status in neonates who screen negative [12]; delayed repeat testing of screen-negative neonates was not performed at either hospital.

The recording of (suspected) infection/sepsis as a cause of jaundice was at the discretion of the clinician completing the LPTR, and was not subject to pre-agreed diagnostic rules (e.g., laboratory confirmation).

At both facilities, gestation was usually reported as ‘post-term’,’term’, ‘preterm’ or sometimes as a specified gestational week or range of weeks. The recorded data was used to classify neonates as ‘Term’ (≥37 weeks’) or ‘Preterm’ (<37 weeks’); where a range of weeks was recorded (e.g., “36-38 weeks’”), classification was based on the midpoint (e.g., 37 weeks’).

### Analysis

In the first stage of analysis, we simply described the characteristics of the treated neonates in each of the hospitals, with no statistical testing performed. The second stage compared characteristics of two sub-groups of neonates’ treated for jaundice: those presenting with ABE, and those not recorded as having ABE at hospital admission. The statistical significance of the difference in proportions was assessed using Fisher’s Exact Test for categorical variables, and the Kruskal-Wallis test for a difference in medians in continuous variables. These comparison were performed separately for each hospital.

In the final stage of analysis, we aggregated data from the two hospitals and used multiple logistic regression to assess the joint effect of multiple risk factors, restricted to variables that were not considered to be intervening variables; TSB at presentation and poor feeding at presentation were both excluded for this reason. The analysis followed a pre-specified modelling strategy: [13] all variables statistically significant at *p* < 0.20 in univariate models were screened in the multivariate model and retained if significant at *p* = 0.05; first order interaction terms between significant main effects were assessed and retained if statistically significant; and the fit of the final model was assessed using the area under the ROC curve (‘c-statistic’) and the Hosmer-Lemeshow Goodness of Fit test. Analysis was undertaken in SAS v9.4.

## Results

Individual patient data was collected for 251 neonates treated on LED phototherapy machines, over 20 consecutive months, at Hospital A (from December 2011 to July 2013, inclusive), and for 339 neonates treated over 13 *non*-*consecutive* months at Hospital B (from December 2011 to 30 June 2013, but with no data provided for February 2012, May to July 2012, and for January and February 2013). The average number of neonates treated per complete month was 13/month (Interquartile Range [IQR] of 4–32) at Hospital A, and 30/month (IQR: 11–46) at Hospital B.

### Characteristics of treated neonates

Table 1 presents key characteristics of neonates treated with LED phototherapy. Both hospitals report a slight preponderance of male patients (57 % at Hospital A vs 59 % at Hospital B). Home birth and self-referral to hospital were not uncommon, affecting around a quarter of treated patients at each hospital, while phototherapy prior to hospital admission was relatively rare (11 % at both hospitals). The hospitals differed in two important ways: Hospital A had a lower proportion of infants admitted weighing <2,500 g (27 %) than Hospital B (42 %); and the reported incidence of ABE at admission was lower at Hospital A (13 %) than Hospital B (21 %).

**Table 1:** Characteristics of LED phototherapy patients

	Hospital A	Hospital B
	*n* = 251	*n* = 339
Percentage male: n (%)^a^	142 (56.6 %)	195 (58.9 %)
Place of birth: n (%)		
Home: n (%)	78 (31.1 %)	88 (26.0 %)
Hospital/Clinic: n (%)	160 (63.7 %)	237 (69.9 %)
Other/Not stated: n (%)	13 (5.2 %)	14 (4.1 %)
Source of referral: n (%)		
Home: n (%)	54 (21.5 %)	90 (26.6 %)
Hospital: n (%)	40 (15.9 %)	212 (62.5 %)
Clinic: n (%)	103 (41.0 %)	12 (3.5 %)
Other/Not stated: n (%)	54 (21.5 %)	25 (7.4 %)
Phototherapy elsewhere, prior to admission: n (%)	28 (11.2 %)	34 (11.0 %)
Admission weight (g)^c^: Median (IQR)	2,800 (2,400-3,200)	2,600 (1,940-3,040)
< 1,000 g: n (%)	2 (0.8 %)	2 (0.6 %)
1,000-1,499 g: n (%)	5 (2.0 %)	29 (8.8 %)
1,500-2,499 g: n (%)	61 (24.5 %)	107 (32.5 %)
2,500 g +: n (%)	181 (72.7 %)	191 (58.1 %)
ABE^d^ noted at admission		
No: n (%)	219 (87.3 %)	267 (78.8 %)
Yes: n (%)	32 (12.7 %)	72 (21.2 %)

*n* number, *IQR* Interquartile range (25^th^ percentile – 75^th^ percentile)

^a^Gender missing for 8 records at Hospital B

^b^Phototherapy elsewhere, prior to admission missing for 31 records at Hospital B

^c^Admission weight missing for two records at Hospital A, and 10 records at Hospital B

^d^The term ‘kernicterus’ is routinely used in both hospitals – this has been re-classified as ABE

Table 2 shows relevant information at the start of phototherapy treatment. The median age at the start of phototherapy was 3 days (IQR: 2–5 days) at both hospitals, and median TSB at the start of phototherapy was also similar (311 μmol/L at Hospital A vs 325 μmol/L at Hospital B). Both prematurity and previous sibling treated with phototherapy were less common among treated neonates at Hospital A (15 and 0.4 % respectively) than at Hospital B (26 and 19 %). Significant bruising was rarely recorded in both hospitals, while breastfeeding and feeding poorly was reported in 10 % of treated neonates in Hospital A, but not reported in Hospital B. Infection was the commonest suspected cause of jaundice (63 in Hospital A and 54 % in Hospital B), followed by G6PD deficiency (36 and 17 %) and ABO incompatibility (17 and 15 %); Rhesus incompatibility was rarely recorded (2 and 1 %).

**Table 2:** Information available at the start of LED phototherapy

	Hospital A	Hospital B
	*n* = 251	*n* = 339
At start of phototherapy		
Age (days): Median (IQR)	3.0 (2.0-5.0)	3.0 (2.0-5.0)
TSB (μmol/L)^a^: Median (IQR)	311 (224–445)	325 (234–473)
Risk factors for hyperbilirubinaemia		
Preterm (<37 weeks’)^c^: n (%)	38 (15.3 %)	85 (25.5 %)
Previous sibling treated: n (%)	1 (0.4 %)	66 (19.5 %)
Significant bruising: n (%)	2 (0.8 %)	9 (2.7 %)
Breastfeeding and feeding poorly: n (%)	25 (10.0 %)	Unclear
Suspected cause (multiple selections permitted)		
Suspected infection: n (%)	158 (63.0 %)	183 (54.0 %)
G6PD deficiency^d^: n (%)	91 (36.3 %)	58 (17.1 %)
ABO incompatibility: n (%)	43 (17.1 %)	52 (15.3 %)
Rhesus incompatibility: n (%)	4 (1.6 %)	2 (0.6 %)
Not specified: n (%)	14 (5.6 %)	32 (9.4 %)

n number, IQR Interquartile range (25^th^ percentile – 75^th^ percentile)

^a^TSB at admission missing for 31 records at Hospital A, and seven records at Hospital B

^c^Gestation at admission missing for one record at Hospital A, and six records at Hospital B

^d^Screening test performed, and assessed as screen positive

Table 3 shows key outcomes for treated neonates. The rate of Exchange Transfusion among treated neonates was high in Hospital A (15 %) and markedly higher in Hospital B (26 %). Mortality was also high (7 % at Hospital A vs 11 % at Hospital B). At each facility, 5 infants were noted to have been discharged with CBE, representing around 2 % of treated infants.

**Table 3:** Outcomes of neonates treated with LED phototherapy

	Hospital A	Hospital B
	n = 251	n = 339
Exchange transfusions^a^		
n (%)	38 (15.3 %)	88 (26.0 %)
TSB (μmol/L) at ET: Median (IQR)^*b*^	474 (416–539)	500 (475–513)
Duration of treatment (days): Median (IQR)^c^	2.0 (1.0-2.0)	1.0 (1.0-2.0)
Discharge status		
Discharged: n (%)	221 (88.1 %)	276 (81.4 %)
Removed by family: n (%)	3 (1.2 %)	8 (2.4 %)
Died: n (%)	18 (7.2 %)	38 (11.2 %)
Transferred to other hospital: n (%)	0 (0.0 %)	2 (0.6 %)
CBE^d^: n (%)	5 (2.0 %)	5 (1.5 %)
Not stated: n (%)	4 (1.6 %)	10 (3.0 %)

*n* number, *IQR* Interquartile range (25^th^ percentile – 75^th^ percentile)

^a^At Hospital A, data missing on exchange transfusion status of three records

^b^TSB at ET missing for 12 transfused neonates at Hospital A and for 42 transfused neonates at Hospital B; note that most readings at Hospital B were performed on equipment with a maximum reading of 513 μmol/L

^c^Duration of treatment unavailable for 22 records at Hospital A and 84 records at Hospital B

^d^‘Kernicterus’ is the term routinely used in both hospitals; in this situation it is assumed to refer to CBE

### Comparing characteristics of neonates with and without ABE at admission

Table 4 compares neonates presenting with and without ABE for age at admission to hospital and TSB at admission. At both hospitals, neonates admitted with ABE had a statistically significantly higher median TSB at admission (694 vs 291 μmol/L at Hospital A, and 496 vs 287 μmol/L at Hospital B; both with *p* < 0.0001). At both hospitals, the median age of neonates admitted with ABE was 4 days, in comparison to 3 days for jaundiced neonates admitted without ABE (*p* = 0.27 at Hospital A, and *p* = 0.10 at Hospital B).

**Table 4:** Age and TSB at admission of neonates with and without ABE at admission

	Hospital A					Hospital B				
	ABE at admission (*n* = 32)		No ABE at admission (*n* = 219)		p-value	ABE at admission (*n* = 72)		No ABE at admission (*n* = 267)		*p*-value
	n	Median [IQR]	n	Median [IQR]		n	Median [IQR]	n	Median [IQR]	
Age at start of phototherapy (days)	32	4.0 (2.0-5.0)	219	3.0 (2.0-5.0)	0.27	72	4.0 (2.0-5.5)	267	3.0 (2.0-5.0)	0.10
Total serum bilirubin at admission^a^(μmol/L)	28	694 (581–795)	192	291 (211–401)	<0.0001	69	496 (475–513)	263	287 (210–393)	<0.0001

*n* number, *IQR* Interquartile range (25^th^ percentile – 75^th^ percentile)

^a^The majority of readings at Hospital B were performed on equipment with a maximum reading of 513 μmol/L

Table 5 shows the discharge status of neonates with and without ABE at admission. ABE at hospital presentation was the key predictor of poor outcome at both hospitals. At Hospital A, 15 of 32 (47 %) neonates with ABE at presentation died, and an additional five (16 %) survived with CBE; this compares to three deaths and no CBE at discharge among the 219 neonates who did not have ABE at admission (1.4 %). At Hospital B, 18 of 72 neonates (25 %) admitted with ABE died, and an additional 5 neonates (7 %) survived with kernicterus; this compares to 20 deaths (7 %) and no survivors with kernicterus among neonates admitted without ABE.

**Table 5:** Selected characteristics and outcomes of neonates with and without ABE at admission

		Hospital A					Hospital B				
		ABE at admission (*n* = 32)		No ABE at admission (*n* = 219)		p-value	ABE at admission (*n* = 72)		No ABE at admission (*n* = 267)		*P*-value
		n	%	n	%		n	%	n	%	
Demographic	Male^a^	17	53.1	125	57.1	0.71	45	62.5	150	57.9	0.50
	Home birth	22	68.8	56	25.6	<0.0001	38	52.8	50	18.7	<0.0001
	Referred from home	16	50.0	38	17.4	0.0001	40	55.6	50	18.7	<0.0001
Selected risk factors for hyperbilirubinaemia	Preterm (<37 weeks’)^b^	3	9.4	35	16.1	0.43	6	8.3	79	30.3	<0.0001
	Previous sibling	0	0.0	1	0.5	1.0	15	20.8	51	19.1	0.74
	Significant bruising	1	3.1	1	0.5	0.24	3	4.2	6	2.3	0.41
Previous phototherapy		2	6.3	26	11.9	0.55	9	12.5	25	10.6	0.67
Suspected cause (multiple selections permitted)	Suspected infection	23	71.9	135	61.6	0.32	34	47.2	149	55.8	0.23
	G6PD deficiency^c^	14	43.8	77	35.2	0.43	29	40.3	29	10.9	<0.0001
	ABO incompatibility	3	9.4	40	18.3	0.31	8	11.1	44	16.5	0.36
	Rhesus incompatibility	0	0.0	4	1.8	1.0	0	0.0	2	0.8	1.0
	Other/Not specified	3	9.4	11	5.0	0.40	4	5.6	28	10.5	0.26
Discharge Status	Died	15	46.9	3	1.4	<0.0001	18	25.0	20	7.5	<0.0001
	Lived, with ABE	5	15.6	0	0.0		5	6.9	0	0.0	
	All other discharge	12	37.5	216	98.6		49	68.1	247	92.5	

*n* number, *IQR* Interquartile range (25^th^ percentile – 75^th^ percentile)

^a^Gender missing for 8 records at Hospital B (none of whom had ABE at presentation)

^*b*^Gestation missing for two records at Hospital A, and six records at Hospital B (none of the eight had ABE at presentation)

^*c*^Screening test performed, and assessed as screen positive

Table 5 also compares neonates with and without ABE at admission for a variety of risk factors for jaundice, and suspected causes of jaundice. At Hospital A, the only statistically significant univariate risk factors for presentation with ABE were home birth and direct referral from home (both *p* < 0.0001). At Hospital B, home birth and self-referral were again significant, and screening positive for G6PD deficiency was also a risk, while preterm birth was protective (*p* < 0.0001 for all factors).

### Multivariate regression model

In the merged dataset the final multivariate model (n = 582 after exclusion of 8 records without data on prematurity) identified the following independent risk and protective factors: home birth, OR_adj_ = 2.3 (95 % CI: 1.04-5.4); self-referral, OR_adj_ = 2.6 (95 % CI: 1.2-6.0); prematurity, OR_adj_ = 0.40 (95 % CI: 0.18-0.85); and a significant interaction between hospital and screening status because screening positive for G6PD deficiency was a strong and significant risk factor at Hospital B (OR_adj_ = 5.9; 95 % CI: 3.0-11.6), but not at Hospital A (OR_adj_ = 1.1; 95 % CI: 0.5-2.5). 

## Discussion

In 2002, the American National Quality Forum defined “Death or serious disability (kernicterus) associated with failure to identify and treat hyperbilirubinemia in neonates” as a serious reportable event [14], reflecting its belief that kernicterus should be a ‘never-event’ that can be entirely avoided. The weak systems of perinatal care that are typical in low resource settings, however, create additional challenges on the path to making kernicterus a ‘never-event’. The reality in low resource settings is that neonates frequently arrive in paediatric emergency rooms with signs of ABE. Numerous studies have demonstrated higher risks of ABE or kernicterus among outborn neonates in low resource settings [15–18]; the current study seeks to add to this, by identifying specific risk factors which lead to these outborn neonates being admitted with established signs of ABE.

### Risks and protective factors for admission with ABE in the current study

#### Home-birth or self-referral

Home born and self-referred neonates are substantially overlapping groups, with 80 % of home births and only 3 % of facility births being self-referred, so it was surprising that both were retained in the final model. We are aware of one study that examined home birth and found no study reporting on self-referral as risk factors for presentation with ABE: a Baghdad study during a period of severe health system disruption found that home birth was not a significant risk factor for CBE or death (OR = 1.2; 95 % CI: 0.6-2.5); the study did not report risk of ABE at presentation [10].

It is unclear that something intrinsic to place of birth would make urban or peri-urban home birth a risk factor for presentation with ABE, so it seems more plausible that home birth is a proxy for other factors. Plausible factors include: characteristics of mothers that give birth at home (e.g., racial differences correlated with both genetic risk and socio-economic status); characteristics of the accoucher (e.g., Traditional Birth Attendants vs midwives) and/or characteristics of the quality of care (e.g., high rates of asphyxia, lower rates of provision of high quality post-partum education about signs requiring referral to hospital); characteristics of the post-partum follow-up (e.g., less frequent follow-up of home births); and persistence of barriers to facility access for both delivery and care of the sick neonate (e.g., physical, attitudinal, or financial). Self-referral, on the other hand, may be a proxy for inaccessibility of facility-based care and/or delayed access for other reasons. The fact that both factors remain significant in the final model, despite substantial overlap, suggests a complex interplay of factors resulting in late presentation.

While it is clear from the prevalence of ABE at admission that *late* presentation is important, it is not clear from our data that age at start of phototherapy is associated with ABE at presentation. Age at presentation was measured crudely in the current study (in days rather than hours, for practical reasons) and that this introduced random error which results in a bias towards statistically non-significant findings, despite the fact that neonates with ABE presented at a median of 4 days, compared to a median of 3 days for neonates without ABE. It is plausible that ‘late’ presentation may need to be defined in terms of disease progression, as well as in chronological terms.

#### G6PD deficiency

G6PD deficiency has been identified as suspected cause of ABE at admission in about a third of the babies in two Nigerian case series of neonates with ABE [15, 19], slightly lower than the rate found in neonates with ABE at Hospitals A and B (44 and 40 % respectively). The prevalence of G6PD deficiency in Myanmar is estimated at 6.1 % [20], somewhat higher than the median of 3.95 % (IQR: 3.4 %- 7.9 %) in 184 countries for which there are estimates [21], but substantially lower than the 16.9 % estimated in Nigeria [20]. On the face of it, one would therefore expect Nigerian studies to have a higher prevalence of neonates with ABE that screen positive for G6PD deficiency. A separate Nigerian case control study found that 67-75 % of neonates with ABE screened positive for G6PD deficiency, in comparison to only 17-22 % of neonates without ABE (range provided as some neonates not screened) [18]. In the current study, G6PD deficiency was only a risk factor for ABE at Hospital B (40 % in neonates with ABE vs 11 % in neonates without ABE). It is unclear why G6PD deficiency was not a risk factor in Hospital A: Hospital A had a higher overall rate of G6PD deficiency (36 % vs 17 % in Hospital B) and a lower overall rate of ABE at presentation (13 % vs 21 %); one possibility is that there are differences in the distribution of local genetic variants causing G6PD deficiency which are in turn related to the incidence of ABE.

#### Prematurity

Several facility-based studies have noted that premature or low birth weight babies have *lower* rates of ABE at presentation: a Nigerian study found that 14 % of cases with ABE weighed <2,500 g in comparison to 37 % of controls admitted to the nursery for any reason (*p* = 0.01) [15]; a separate Nigerian study found that 22 % of cases with ABE weighted ≤2,500 g, in comparison to 42 % of clinically jaundiced neonates admitted to the nursery (*p* = 0.46) [18]; and a Bangladeshi study found that 11 % of neonates that presented with or developed ABE during the study were preterm, in comparison to 21 % of neonates admitted to the nursery with jaundice or who developed jaundice during their stay (*p* = 0.55) [22].

Conversely, low gestation has long been identified as an epidemiological risk factor for kernicterus. For example, a classic 1950–54 study estimated the incidence of kernicterus by gestation among neonates admitted to the newborn units and surviving to 48 h: ≤30 weeks 10,100/100,000 admitted survivors; 31–32 weeks 5,700/100,000; 33–34 weeks 3,200/100,000; 35–36 weeks 1,100/100,000; and ≥37 weeks 800/100,000 admitted survivors [23]. Given this long established risk-factor status, the apparent protective role of low gestation found in the current study, and in the other studies reported above, is interpreted as an artefact arising from the use of controls admitted to the neonatal nursery. The term neonates in these studies are drawn from a pool at extremely high risk of ABE, whereas the preterm neonates are all likely to be routinely admitted and often receive prophylactic phototherapy, simultaneously preventing the development of ABE.

### Other risk factors for ABE

#### Rh (D) isoimmunisation

The incidence of kernicterus in Greece prior to the introduction of exchange transfusion or phototherapy has been estimated at 40/100,000 live births among neonates affected by Rh haemolytic disease [24], while a recent modelling study estimated that roughly one third of ABE cases were caused by Rh disease [21]. In Myanmar, the prevalence of Rh negative blood group prevalence is estimated at 0.8 %, substantially lower than the median of 3.7 % (IQR: 3.5 %-10.0 %) for 138 countries with a neonatal mortality rate above 5/1,000 live births [21]. This low prevalence in Myanmar results in few neonates with ABE due to Rh (D) incompatibility, despite the lack of systematic screening and prophylaxis to prevent Rh (D) isoimmunisation.

#### Sepsis

It has been noted that sepsis can increase the risk of severe hyperbilirubinaemia and or bilirubin neurotoxicity by altering the binding affinity of albumin [25], and that sepsis can result in acute, severe hyperbilirubinaemia [26]. Investigation of this factor in the current study was hampered by the lack of a firm definition, or laboratory confirmation. At Hospital A, 63 % of treated neonates had ‘suspected sepsis’ recorded as a cause while at Hospital B the figure was slightly lower at 54 %; infection/sepsis was not a statistically significant predictor of ABE at presentation in either hospital. The literature on the role of sepsis as a risk factor for ABE in low resource settings is limited: a Nigerian study attributed sepsis as the cause of ABE in 43 % of cases but did not give the rate of sepsis in a control series [15].

### Limitations

The current study was conducted as operational research, resulting in a number of limitations. First, the cohort studied represents neonates treated on LED phototherapy. Participating clinicians state that most infants were treated on the LED machines, and those that were not tended to have lower TSB. Any bias is therefore likely to be small and, if there is bias, it is likely to result in a compressed patient spectrum towards higher risk infants, shrinking risk estimates towards the null.

Second, there were a number of measurement-related limitations: ABE was not formally defined, but relied on the clinical judgement of experienced clinicians at each facility, leaving open the possibility of under- or over-diagnosis of ABE; age at admission was measured in days rather than hours; there was no delayed re-test of neonates that received a negative G6PD screening result; there were no strict definitions of sepsis as a suspected cause of jaundice; different hospitals may have different proclivities to record particular risk factors (e.g., previous sibling treated with phototherapy and significant bruising); and the in-unit desktop Bilirubinometer at Hospital B, used for the majority of TSB readings, was restricted to an upper limit of 513 μmol/L. All of these limitations result in misclassification of risk factors for ABE, or of ABE itself, and this misclassification necessarily results in a bias towards the null (i.e., OR = 1.0). Care should therefore be taken not to over-interpret *non-significant* findings, but statistically significant risk factors can be considered to be robust estimates, possibly with the risk factor status diminished due to misclassification errors.

## Conclusions and next steps

This study demonstrates that home birth and self-referral to a paediatric hospital and, in one hospital screening positive for G6PD deficiency, are significant risk factors for paediatric hospital admission of jaundiced neonates with ABE in Myanmar. Additional information is required to identify possible points at which it is feasible to intervene, to reduce clinically late presentation of outborn neonates with ABE.

Prospective case–control studies have been initiated to identify in more detail the barriers to timely presentation of jaundiced neonates. Depending on the findings of these case–control studies, a range of interventions are possible. Examples of possible interventions include: general information for the community (e.g., to ensure that mothballs are removed from the home environment of neonates to prevent triggering G6PD deficiency); training and information to be distributed by healthcare providers (public and private) to ensure new mothers and their families can identify jaundice, and are aware of the need to self-refer quickly if a baby becomes jaundiced in the first 72 h, or if severe jaundice appears rapidly at any time thereafter; home visits by nurses/midwives in the first week of life, in line with national policy, could be optimally-timed, targeting home-births; training of staff at primary healthcare settings to identify and rapidly transfer babies at risk of exchange transfusion, and development of protocols to ensure rapid transfer of these neonates; and provision of TSB testing equipment and high quality phototherapy equipment to lower level facilities. While the provision of TSB screening equipment to home visit staff is not currently feasible due to cost, new systems such as ‘Bilistick’ costing €150 for the prototype and a few cents per test have been found to be highly accurate [27], raising the possibility that screening can be undertaken at lower levels in the health care system.

Given the many demands on health systems in low-resource settings, it is important to prioritise interventions with a goal of achieving optimal results from any given resource input. The data presented in this report, represent a first step in the process of identifying the interventions required to address the mortality and morbidity associated with ABE in Myanmar, as part of the process of intervening to reduce that mortality and morbidity.

## Abbreviations

ABE: Acute Bilirubin Encephalopathy
BOL: Breath of Life Program
CI: Confidence Interval
CP: Cerebral palsy
CBE: Chronic Bilirubin Encephalopathy
INGO: International Non-Governmental Organization
MTTS: Medical Technology Transfer Services, Hanoi, Viet Nam
NCU: Neonatal Care Unit
NS: Not statistically significant
OR: Odds Ratio
OR_adj_: Adjusted Odds Ratio
TSB: Total Serum Bilirubin
